# The identification and appraisal of assessment tools used to evaluate metatarsus adductus: a systematic review of their measurement properties

**DOI:** 10.1186/s13047-018-0268-z

**Published:** 2018-06-01

**Authors:** Nicole Marshall, Emily Ward, Cylie M. Williams

**Affiliations:** 1 University of South Australia, School of Health Science, Adelaide, SA 5000 Australia; 2 Department of Physiotherapy, Monash University, Frankston, VIC 3199 Australia; 3 Peninsula Health, Allied Health, Frankston, VIC 3199 Australia

**Keywords:** Metatarsus adductus, Assessment, Paediatric, Child

## Abstract

**Background:**

Metatarsus adductus is the most common congenital foot deformity in newborns. It involves adduction of the metatarsals at the Lisfranc joint. A systematic literature review was conducted to investigate the following question: What tools are used to identify and quantify metatarsus adductus and how reliable, valid and responsive are they?

**Methods:**

The following electronic databases were searched for studies describing tools for the identification and quantification of metatarsus adductus in adults and children published from inception to June 2016: Ovid MEDLINE, Embase, CINAHL, Scopus, Web of Science and AMED. Two researchers initially searched all articles by screening titles and abstracts. If there was any doubt as to an article’s eligibility, the full text paper was retrieved. Reference lists and citations of all retained studies were examined in an attempt to locate further studies. Articles were excluded if they were not in English or described other congenital foot conditions that did not include metatarsus adductus. Studies included in the review reporting measurement properties of measurement tools were critically appraised using the Consensus-based Standards for the selection of health Measurement Instruments (COSMIN) critical appraisal tool.

**Results:**

There were 282 articles screened by title and abstract and 28 articles screened from full text. Fifteen articles were included and nine had data that were extractable for appraisal using the COSMIN critical appraisal tool. Techniques to measure metatarsus adductus included the heel bisector method, photocopies, ultrasound, footprints, dynamic foot pressure and radiographs. There was a paucity of quality data reporting the reliability, validity or responsiveness for measuring metatarsus adductus. Several radiographic angles showed good reliability (intraclass correlation (ICC) – 0.84, 0.97) in adults during pre-operative planning.

**Conclusion:**

There have been multiple assessment techniques proposed for quantification of metatarsus adductus, but there is paucity of reliability, validity or responsiveness to measurement data about these techniques, especially in relation to the paediatric population. Further consideration of measurement testing is required to determine if the most common non-radiographic measures of metatarsus adductus are acceptable for clinical use.

**Electronic supplementary material:**

The online version of this article (10.1186/s13047-018-0268-z) contains supplementary material, which is available to authorized users.

## Background

Metatarsus adductus is the most common congenital foot deformity in newborns with a reported prevalence of one to two cases per 1000 births [1]. The deformity appears as an adduction or medial deviation of the forefoot at the tarsometatarsal joints (Lisfranc joint) with associated soft tissue contractures that may lead to osseous changes over time [2–4]. The metatarsals are deviated medially in the transverse plane resulting in a convex lateral border of the foot and a prominent styloid process [3, 5, 6]. Metatarsus adductus can be differentiated from other congenital foot conditions as it is purely a forefoot condition that does not involve the hindfoot unlike talipes equinovarus or skewfoot [3]. The exact aetiology of metatarsus adductus is unknown, however, it has been suggested that increased intrauterine pressure, osseous abnormality and abnormal muscle attachments may be potential causes [7–12]. Between 87 and 90% of flexible metatarsus adductus cases resolve spontaneously without the need for further treatment [10, 11, 13, 14].

There is inconclusive evidence on the long term effect of metatarsus adductus on the adult foot [15]. Some authors propose that if metatarsus adductus persists into adulthood it can lead to the development of hallux valgus, skewfoot or hammer toes, intoeing, increased medial tibial torsion, fifth metatarsal stress fractures, difficulty fitting into shoes and can contribute to increased falling or tripping later in life [7, 12, 16–18].

Metatarsus adductus is commonly diagnosed according to the presence and severity of the deformity and the degree of flexibility using the Bleck manual assessment [3, 4, 19–21]. Clinically, assessments are used to determine if treatment is required, the method of treatment, monitoring progress and to ensure the child’s foot has returned to a normal foot alignment post intervention [22]. Whilst there are numerous assessment techniques published to quantify the presence of metatarsus adductus, there is little research assessing validity, reliability or responsiveness of these measures. This is potentially problematic for clinicians, as quantification of the condition drives treatment and management decisions [15].

There have been several assessment techniques proposed in the literature for the assessment of the deformity to quantify the severity. In 1983, Bleck created a visual assessment called the ‘heel bisector method’. It involves firstly visually identifying the severity of the deformity and categorising it as mild, moderate or severe according to the heel bisector and secondly making a classification of flexibility as fully flexible, partially flexible or severe. This simple method requires minimal time for both the clinician and patient and as it was observation based, and cost effective [3]. This measure has been modified and used in numerous studies since its development and continues to be clinically utilised to reassure parents that their child’s foot position will resolve with time or to drive treatment initiation [4, 19, 20].

Other assessment methods described within the literature to grade severity include a multitude of radiographic angles [23–25], photocopies of the child’s foot [19] and the V-finger test [16, 17]. Radiographs may be considered time consuming and unnecessary given potential risk to the child. Photocopying the child’s foot poses potential risk due to glass breakage. Recent studies suggest the use of ultrasound as a measurement technique as it allows for the imaging of cartilaginous structures [21].

As previously discussed, many treatment options are initiated based on the observed presence of deformity, to measure severity and flexibility of the condition. Therefore if the tool used to measure these components was not adequate, this has implications for the initiation of treatment where needed, or potentially unnecessary treatment leading to a potential poor prognosis for that child. [16]. The primary aim of this systematic review was to identify, and where possible, appraise the measurement properties of all known methods for identifying the severity and/or flexibility of metatarsus adductus.

## Methods

### Search strategy

The PRISMA guidelines for systematic reviews were followed [26]. The question and search terms were developed using the broad concepts of the PICO (Population, Intervention Comparison and Outcomes) model [27]. The systematic review was registered with Prospero (CRD42016039622). The following electronic databases were searched for studies describing assessment tools for the identification and quantification of metatarsus adductus in adults and children published from inception to June 2016: Ovid MEDLINE, CINAHL, Scopus, Web of Science and AMED. Broad MeSH terms and keywords were used to identify the articles of interest (e.g. Metatarsus adductus) and quantification terminology (e.g. measure, assess). Additional file 1 contains the full search terms and truncation used within each database. Citation chaining was undertaken to identify any articles that may have been missed in the initial search strategy. It involved employing a forward and backward searching strategy using Google Scholar citations to identify relevant articles using a single paper as a starting point which creates a ‘chain’ of references linked backward and forward from the original article. The inclusion and exclusion criteria used for search strategy is listed in Table 1.

**Table 1:** Inclusion and exclusion criteria for articles included in the systematic review

Inclusion Criteria:	Exclusion Criteria:
• Studies describing a tool measuring metatarsus adductus.	• Studies describing congenital foot deformities with no inclusion of metatarsus adductus These included but were not limited to: skewfoot, congenital talipes equinovarus, metatarsus primus varus, congenital metatarsus varus or serpentine foot.
• Studies reporting any measurement properties of measurement tools for assessing metatarsus adductus as defined by the COSMIN tool. • All study designs • Studies based on both adults and children	• Non-English publications. • Grey literature

**Fig. 1:**
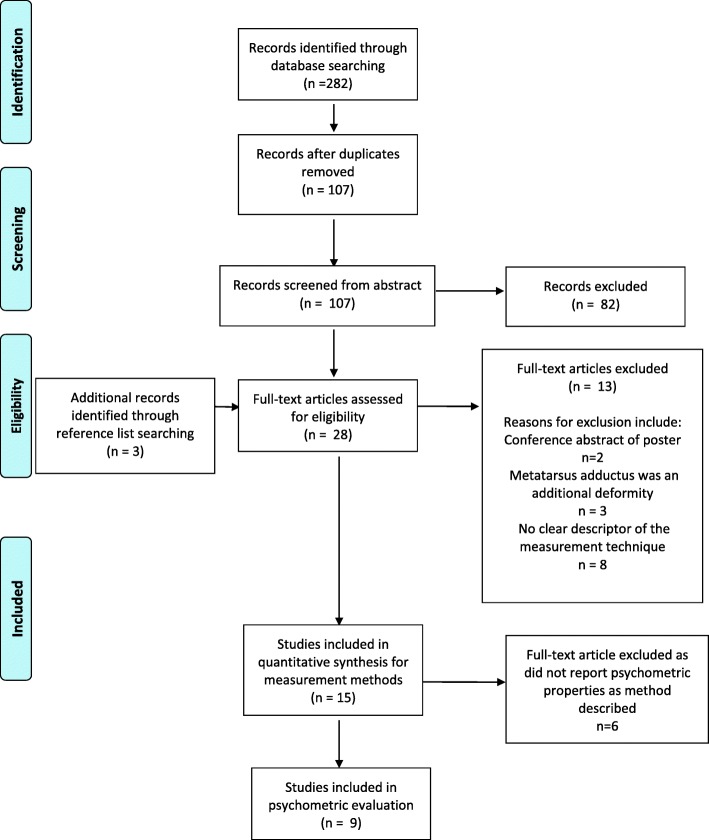
PRISMA flow of article inclusion

Two researchers (NM & CMW) initially screened the title and abstract of all articles. If there was any doubt as to an article’s eligibility, the full text paper was retrieved. Articles were imported into Covidence for screening [28]. The full text of included study abstracts were independently screened against the inclusion criteria by two reviewers (NM & EW). Any disagreement was resolved by discussion or through consultation with the third author. Figure 1 displays the search process.

### Data extraction

Data were extracted by the first author and where there were queries, these were resolved with all authors. Data extracted included the study design, population, assessment measure/description, sample size, reliability (inter/intra rater reliability), statistical quality, available domains of the COSMIN and author derived measurement [29].

### Critical appraisal of study methodology quality

Where studies reported measurement properties, they were critically appraised using the Consensus-based Standards for the selection of health Measurement Instruments (COSMIN) critical appraisal tool [29]. The COSMIN was developed in an international Delphi study to improve the selection of health measurement instruments [29]. This was found to have adequate content validity [30]. COSMIN assessed the internal consistency, reliability, measurement error, hypothesis testing, content and structural validity, responsiveness and interpretability of a measurement [31]. Articles chosen for inclusion in the synthesis were subject to appraisal by two independent reviewers.

The COSMIN may be utilised as a modular tool and used to assess the quality of measurement based studies including patient reported outcome measures, scales and simple measures such as those appraised in this present study. Therefore only relevant parts of the checklist were used for quality evaluation of measurements [29] included: box B (reliability), D (content validity), H (criterion validity) and I (responsiveness). For example, if the study reported on the measurement properties involving reliability then box B was the only part of the COSMIN completed [29]. COSMIN also requires generalizability to be assigned for each measure. The results of these modules were then assessed using a 4 point scale to allow calculation of the overall methodological quality score for each study. This methodological quality score per box was obtained by assigning the lowest rating of any item in a box (‘worse score counts’).

## Results

### Included studies

There were 15 studies included in this review. Of these 15 articles, nine reported on measurement properties that were able to be critically appraised for quality using the COSMIN and six reported methods of measurement that were unable to be appraised. Table 2 provides a description of the 15 included studies. A meta-analysis was not performed as studies were heterogeneous. Each study varied in terms of participant age, country the study was conducted, gender and measurement tool used. Out of the 15 studies, two studies were conducted on an adult population, eight studies had a paediatric population and five studies did not mention age. The majority of studies were cohort study designs (Table 2). There were six methods of measurement described to assess the severity or flexibility of metatarsus adductus (Table 3).

**Table 2:** Studies included within review

Primary Author	Study Design	Country	Sample Size	Gender Male/ Female	Age (Age (SD), Range)	Measure	Inclusion Criteria	Exclusion Criteria	Data collection method	Tool developed by author:
Engel [35]	Retrospective cohort	USA	571 radiographs	Not stated	Not stated	1. Traditional metatarsus adductus angle (intersection of longitudinal axis of lesser tarsus with bisector of second metatarsal) 2. Simplified metatarsus angle (intersection of longitudinal bisector of 2nd metatarsal and medial cuneiform)	Foot radiographs from Oxford hospital.	Not stated	Weight-bearing foot radiographs	Yes
Dawoodi [38]	Literature review	United Kingdom	Not stated	Not stated	Not stated	1. Traditional metatarsus adductus angle (intersection of longitudinal axis of lesser tarsus with bisector of second metatarsal) 2. Engel’s angle (bisection of middle cuneiform with longitudinal axis of second metatarsal) 3. Modified Engel’s angle using the base of the middle cuneiform as a reference line	Not stated	Not stated	Not stated	No
Dawoodi [32]	Validation study	United Kingdom	130 patients (50 randomly selected radiographs)	119/14	50.6 (15.4) years, 14–80 years	1. Sgarlarto’s angle/traditional metatarsus adductus angle (angle between 2nd metatarsal and longitudinal axis of the lesser tarsus using 4th metatarso-cuboid joint as reference) 2. Rearfoot-2nd meta-tarsal angle 3. Engel’s angle (bisection of middle cuneiform with longitudinal axis of second metatarsal) 4. Modified Engel’s angle using the base of the middle cuneiform as a reference line	Symptomatic hallux abductovalgus, listed for corrective surgery	Evidence of trauma, Previous surgery	Dorsoplantar radiographic views.	No
French [34]	Non-Controlled Trial	USA	42 (68 ft)	29/13	9.1 months 1–3 years	1. Lateral calcaneal 5th Metatarsal angle (line representing the lateral aspect of calcaneus and the longitudinal bisector of the fifth metatarsal) 2. Inter-metatarsal angle (angle formed by longitudinal bisectors of 1st and 2nd metatarsals) 3. Talus first metatarsal angle (longitudinal bisectors of talus and the first metatarsals) 4. Talocalcaneal angle (Kite’s angle) (longitudinal bisector of the talus and the calcaneus) 5. First metatarsal fifth metatarsal angle (angle formed by longitudinal bisection of 1st and 5th metatarsals) 6. Talocaneal angle lateral view (bisection of talus and a line representing inferior aspect of the calcaneus)	Clinical observation of metatarsus adductus	Not stated	AP and lateral radiographs	First metatarsal -5th metatarsal angle only was author driven.
Hutchinson [39]	Expert Opinion	USA	Not stated	Not stated	Not stated	Describes Kite’s talocalcaneal angle and calcaneal metatarsal angle without labeling of method	Not applicable	Not applicable	Radiographs	No
Widhe [13]	Prospective cohort	Sweden	2, 401	Not stated	0–16 years	Dynamic foot pressure and gait analysis	Not specifically stated.	Not stated	EMED dynamometric system	No
Knörr [4]	Prospective cohort	Spain	26 (34 ft)	16/10	5.7 years, 3–8.5 years	1. Heel bisector method (undescribed) 2. First cuneiform metatarsal angle (medial angulation of first cuneometatarsal joint) 3. Metatarsal-metaphyseal angle (metatarsal metaphyseal incurvation)	Rigid Metatarsus adductus scheduled for surgery	Not stated	Weight bearing radiographs	No
Miron [21]	Expert Opinion	Canada	N/A	Not stated	Not stated	Lateral displacement of the medial (first) cuneiform over the navicular as the single feature.	Not applicable	Not applicable	Ultrasonography	Yes
Lepow [25]	Retrospective cohort	USA	15	7/8	6 months, (2–15 months)	1. Traditional metatarsus adductus angle (intersection of longitudinal axis of lesser tarsus with bisector of second metatarsal).	Diagnosed with metatarsus adductus	No treatment No radiography	WB AP radiographs	No
Herzenberg [20]	Prospective cohort	USA	27 (43 ft)	Not stated	3–9 months.	1. Footprints analysed using modification of Bleck’s heel bisector method. A transparent template with longitudinal bisector was applied over footprint and graded 2. Talocalcaneal angle (Kite’s angle) (longitudinal bisector of the talus and the calcaneus)	< 9 months, Failed program of parent stretching	Children older than 9 months of age	Footprints	Yes
Smith [19]	Expert Opinion	USA	Not applicable	Not applicable	Not applicable	Heel bisector drawn onto photocopy of a child’s footprint. A second copy in maximum correction for quantification of flexibility	N/A	N/A	Photocopied footprint	Yes
Dominguez [33]	Retrospective cohort	Spain	121 (20 randomly selected radiographs)	106/103	23.88 years (2.85), 20–29 years	Metatarsus adductus angle - cuboid and the 4th metatarsal and cuboid and the 5th metatarsal	> 20 years No history of foot surgery or alteration in plantar pressures distribution	Disease or trauma causing foot pathology, pain, toe deformities	Dorsoplantar radiographs weight bearing	No
Cook [23]	Retrospective cohort	USA	40 ft	Not stated	9 weeks – 9 months	Berg classification - talus-first metatarsal deviation, calcaneal line to cuboid, AP talocalcaneal line, lateral talocalcaneal angle	Metatarsus adductus diagnosis on clinical examination	Poor quality radiograph	Radiographs	no
Berg [2]	Prospective cohort	USA	84	50/34	2.5 years 2.2–3.8	Talocalcaneal angle (Kite’s angle) (longitudinal bisector of the talus and the calcaneus)	Stretches failed to resolve metarasus adductus	Neurological conditions	AP weight-bearing radiographs	Yes
Bleck [3]	Retrospective cohort	USA	160	93/67	5–36 months	Heel bisector method: Severity measure: The heel bisector centres of the hindfoot plantar surface. Line crosses between the 2nd and 3rd toes = normal, through the 3rd toe (mild deformity), between 3rd and 4th toes (moderate), between 4th and 5th toes (severe deformity). Flexibility measure: Abduction beyond the midline heel bisector (mild classification), only to the midline (partially flexible), no abduction possible (severe)	Metatarsus adductus diagnosis on clinical examination	Dynamic hallux varus, metatarsus primus varus or serpentine foot.	Observation and physical assessment	Yes

*N/A Not applicable

**Table 3:** Identified measurement techniques for measuring metatarsus adductus

Type of measure:	Frequency of measure *n* = 15
Visual – heel bisector method [3, 4, 19–21, 38]	6 (40%)
Radiographs [2, 4, 20, 23, 25, 32–35, 38, 39]	11 (73%)
Ultrasound [21]	1 (7%)
Photocopier [19]	1 (7%)
Footprints [20]	1 (7%)
Dynamic foot pressure and gait analysis [13]	1 (7%)

### Reliability of measures employed to quantify metatarsus adductus

There were three studies assessing the reliability of three different individual radiographic angles to assess metatarsus adductus [23, 32, 33]. These measures (Table 3) included the traditional and modified metatarsus adductus angle, the Engel and/or modified Engel angles and the rearfoot-second metatarsal angle [32, 33]. Only radiological measures reported any intra-rater reliability intra-class correlation coefficient (ICC). These ranged from 0.85–0.97 and the inter-rater reliability ICC ranged from 0.84–0.972 [32, 33] (Table 4).

**Table 4:** Intra and inter-rater reliability of radiographic angles used to assess metatarsus adductus

Radiographic angles:	Description of measurement:	Study	Intra-rater ICC:	Inter-rater ICC:
Traditional metatarsus adductus angle (5th)	Angle between the second metatarsal and longitudinal axis of the lesser tarsus using the fifth metatarso-cuboid joint as a reference	[32]	0.92	0.87
		[33]	0.970	0.962
Modified metatarsus adductus angle (4th)	Angle between the second metatarsal and longitudinal axis of the lesser tarsus using the forth metatarso-cuboid joint as a reference	[38]	0.91	0.93
		[33]	0.962	0.972
Rearfoot-2nd metatarsal angle	Angle between the longitudinal bisection of the second metatarsal bone and the line parallel to the lateral border of the calcaneum	[32]	0.85	0.87
Engel’s angle	Angle between the longitudinal axis of the middle cuneiform and the longitudinal axis of the second metatarsal	[32]	0.90	0.84
Modified Engel	Angle between the longitudinal axis of the second metatarsal and a line perpendicular to the proximal articular surface of the middle cuneiform and the angle between the rearfoot reference line (line parallel to the lateral border of the calcaneum) and the longitudinal axis of the second metatarsal	[32]	0.92	0.91
The Berg Classification system	Four radiographic measurements that categorises foot deformities into; metatarsus adductus, complex metatarsus adductus, simple skewfoot and complex skewfoot This includes the following: talus-first metatarsal deviation, calcaneal line to cuboid, AP talocalcaneal line, lateral talocalcaneal angle	[23]	Average intra-rater consistency: 74% (ranged from 66.7–81%).	Average inter-rater consistency: 64% (ranged from 61.9–66.7%).

The Berg’s classification system reported diagnostic agreement ranging from 66.7–81% for inter-rater consistency and 61.9–66.7% for intra-rater consistency. During reliability testing, authors removed five radiographs that produced high disagreement between reviewers from results, the administrations were not independent and there was no time interval between reviewing the images.

### Methodology quality of the included studies

The COSMIN criterion was applied to the nine studies reporting measurement properties to determine their methodological quality (Table 5). Three reported the reliability of the measure and assessed as fair to good methodological quality [23, 32, 33]. One included reported content validity which rated poorly for methodological quality [34], one included criterion validity which also rated poorly for methodological quality [35]. There were five of the nine included studies assessed for responsiveness. All five studies were rated poorly for methodological properties relating to responsiveness according to the COSMIN criterion [2, 4, 20, 25, 34]. There were no studies reporting specific data on sensitivity or specificity in any included research.

**Table 5:** COSMIN critical appraisal tool used for studies that could be analysed for their measurement properties

Study:	Measurement tool:	Author driven:	Reliability (B):	Content Validity (D):	Criterion Validity (H):	Responsiveness (I):
Berg et al. [2]	Radiographs: Berg classification system	Yes	n/a	n/a	n/a	Poor
Cook et al. [23]	Radiographs: Berg classification system	No	Poor	n/a	n/a	n/a
Dawoodi et al. [32]	Radiographs: metatarsal angle (4th), modified metatarsal angle (5th), rearfoot – 2nd metatarsal angle, Engel’s Angle, modified Engel’s angle.	No	Good	n/a	n/a	n/a
Dominguez et al. [33]	Radiographs: Traditional metatarsus adductus angle (cuboid and the 4th metatarsal as reference), modified metatarsus adductus angle (cuboid and the 5th metatarsal).	No	Fair	n/a	n/a	n/a
Engel, et al. [35]	Radiographs: Traditional metatarsus adductus angle and the modified metatarsus adductus angle	Modified metatarsus adductus angle only.	n/a	n/a	Fair	n/a
French, et al. [34]	Radiographs: Lateral calcaneal 5th metatarsal angle, inter-metatarsal angle, talus first metatarsal angle, talocalcaneal angle (Kite’s angle), first metatarsal fifth metatarsal angle, talocaneal angle (lateral view).	First-metatarsal fifth-metatarsal angle was author driven. All other angles were not.	n/a	Poor	n/a	Poor
Herzenberg et al. [20]	Footprints analysed using a modified version of Bleck’s measurement.	Yes	n/a	n/a	n/a	Poor
Knörr et al. [4]	Radiographs: First cuneiform metatarsal angle, metatarsal-metaphyseal angle.	No	n/a	n/a	n/a	Poor
Lepow et al. [25]	Radiographs: Paediatric metatarsus adductus angle	Yes	n/a	n/a	n/a	Poor

There were several other measurement techniques identified for assessing metatarsus adductus. These were unable to be critically appraised using the COSMIN due to a lack of reported measurement properties. These measures included Bleck’s heel bisector method [3], photocopying the child’s foot [19], ultrasound [21], and dynamic foot pressure [13].

## Discussion

Metatarsus adductus has been described as the most common congenital foot deformity presenting in newborns [1]. While this condition is self limiting in the vast majority of cases, there is time when treatment is warranted and guided by measurements assessed within this review. Several measurement techniques are reported for assessment of this condition, but there was limited high quality evidence supporting the measurement properties of many of these measurements.

Radiographic angles had high levels of reliability for measuring metatarsus adductus in adults when taken during pre-operative planning. However, these studies should be interpreted with caution as they were conducted with adult participants with no history of metatarsus adductus. Therefore, results may not be transferable to the paediatric population. Furthermore, radiographs measure and quantify the severity of metatarsus adductus but do not take into account the flexibility of the condition. There is little benefit for supporting using this measurement technique in children unless surgical management is recommended [16, 36, 37]. One study assessed radiographic angles in children however, the author developed their own rating tool and it scored poorly for quality on the COSMIN for both content validity and responsiveness [34].

Metatarsus adductus is primarily identified and treated conservatively in the paediatric population where the benefits and risks of measurement technique must be considered [15]. Radiographs and the associated angles measure the osseous deviation and change. In the younger population, the lack of tarsal bone ossification does not allow many of these angles to be easily calculated [21]. The radiographic angles with the highest reliability [32, 33] identified in this review were calculated and reliant on osseous structures that appear after the age of five [38]. Ultrasonography was utilised as a dynamic imaging modality for quantification of metatarsus adductus in infants as it allows for the imaging of the cartilaginous structures. It also differentiates between metatarsus adductus and skewfoot, as in metatarsus adductus the medial cuneiform will be displaced laterally over the navicular [21]. Whilst this is a new imaging modality, no studies have assessed the measurement properties of this method. This has the potential to be a costly measurement technique requiring additional skills by clinicians for interpretation, however, would negate the radiation exposure that radiographs impose.

The Bleck’s heel bisector method was the most frequently reported measurement for assessing metatarsus adductus in the paediatric population. It is the measure that appears most frequently within studies relating to metatarsus adductus [3, 4, 19–21, 38]. The Bleck measurement was also recently used to assess the treatment outcome of stretching for metatarsus adductus versus no treatment [22]. This simple measure requires a short time for the clinician to assess both any flexibility and severity of the adduction deformity. As this measure is a manual assessment that requires no equipment, it is considered simple and is less costly than radiological measurement. No studies have assessed the measurement properties of this measurement. Due to the commonality of this measure and clinical use to guide treatment and success of treatment, further research should be considered to determine the measurement properties of this assessment.

There are a number of limitations within this review that impact findings and recommendations. English only articles were included and due to limited and varied data extracted, no meta-analysis was performed. A broader search encompassing grey literature may have found additional papers reporting the quality of measures. They may have included hospital or department protocols or guidelines. Future studies should consider encompassing these within the review. The generalisability of radiological reliability findings to the paediatric population was not possible due to osseous development and the methodology of measure development did not always include the paediatric population.

Sensitivity and specificity was not specifically mentioned in any of the measurement tools analysed in this review which impacted on the responsiveness criterion of the COSMIN. This also limited the number of items that could be selected from the COSMIN appraisal tool and used to analyse each study. Additionally, there was no cost-benefit analysis of any of the measures found within the literature.

## Conclusion

There have been multiple assessment techniques proposed for quantification of metatarsus adductus but there is a paucity of quality data on reliability, validity or responsiveness of these techniques, especially in relation to the paediatric population. There have been several radiographic measures shown to have good reliability in adult participants and are used for surgical guidance. Further research is required to determine if simple measures commonly guiding reassurance or implementation of conservative treatment are a reliable way of measuring metatarsus adductus in the paediatric population.

## Additional file

Additional file 1: Search terms and truncation used within each database. (DOCX 89 kb)

## Abbreviations

COSMIN: Consensus-based Standards for the selection of health Measurement INstruments
ICC: Intraclass correlation coefficient
IRT: Item response theory
