# Supplementary material for: The identification and appraisal of assessment tools used to evaluate metatarsus adductus: a systematic review of their measurement properties
Source: J Foot Ankle Res. 2018 Jun 1;11:25. doi: 10.1186/s13047-018-0268-z (PMC5984762; doi:10.1186/s13047-018-0268-z)
Supplement: Supplementary file 1 — Search terms and truncation used within each database. (DOCX 89 kb) [file 13047_2018_268_MOESM1_ESM.docx]

**Additional File 1**

Full search terms and truncation used within each database are detailed below.

**CINAHL:**

“metatarsus adductus” or “pes adductus” or “C-shaped foot” or “metatarsus varus”) AND (measur* or quant* or qual* or assess* or bleck or “heel bi?sector” or x?ray or “plain film*” or photocop* or print*) OR (“x-rays” or “radiography) AND (child* or p?ediatric or infant* or neonate* or toddler* or kid* or baby or babies

**Scopus:**

(“metatarsus adductus” or “pes adductus” or “C-shaped foot” or ‘metatarsus varus”) AND (measure* OR quant* OR qual* OR assess* or bleck or “heel bi?sector” or radio* or x?ray or “plain film” or “photocop* OR print*) AND (child* or p?ediatric or infant* or neonate* or toddler* or kid* or baby or babies)

**Web of Science:**

(“metatarsus adductus” or “pes adductus” or “C-shaped foot” or “metatarsus varus” AND (measure* or quant* or qual* or assess* or bleck or “heel bi?sector” or radio* or x?ray or “plain film*” or photocop* or print*) AND (child* or p?ediatric or infant* or kid* or baby or babies)

**Medline:**

(metatarsus adductus or pes adductus or C-shaped foot or metatarsus varus) AND (measur* or quant* or qual* or assess* or bleck or heel bi?sector or radio* or x?ray or plain film* or photocop* or print*) OR (radiography or x-rays/) AND (child/ or infant/ or child* or p?ediatric or infant* or neonate* or toddler* or kid* or baby or babies)

**Embase:**

(metatarsus adductus or pes adductus or C-shaped foot or metatarsus varus) AND (measur* or quant* or qual* or assess* or bleck or heel bi?sector or radio* or x?ray or plain film* or photocop* or print*) OR (radiography or x-rays/) AND (child/ or infant/ or child* or p?ediatric or infant* or neonate* or toddler* or kid* or baby or babies)
